# Combined EphB2 receptor knockdown with radiation decreases cell viability and invasion in medulloblastoma

**DOI:** 10.1186/s12935-017-0409-7

**Published:** 2017-03-29

**Authors:** Shilpa Bhatia, Kellen Hirsch, Sanjana Bukkapatnam, Nimrah A. Baig, Ayman Oweida, Anastacia Griego, Dylan Calame, Jaspreet Sharma, Andrew Donson, Nicholas Foreman, Christopher Albanese, Sujatha Venkataraman, Rajeev Vibhakar, Sana D. Karam

**Affiliations:** 1 Department of Radiation Oncology, University of Colorado Denver, Anschutz Medical Campus, 1665 Aurora Court Suite 1032, Aurora, CO 80045 USA; 2 School of Medicine, University of Colorado Denver, Anschutz Medical Campus, Aurora, CO 80045 USA; 3 Department of Oncology, Lombardi Comprehensive Cancer Center, Georgetown University Medical Center, Washington, DC 20057 USA; 4 Department of Pediatrics and Section of Pediatric Hematology/Oncology/BMT, Children’s Hospital Colorado and University of Colorado Denver, Anschutz Medical Campus, Aurora, CO 80045 USA

**Keywords:** EphB2, Radiosensitization, Medulloblastoma, Invasion

## Abstract

**Background:**

Medulloblastoma is one of the most common types of pediatric brain tumor characterized by the subpopulation of cells that exhibit high invasive potential and radioresistant properties. In addition, dysregulated function and signaling by Eph family of receptors have been shown to impart pro-tumorigenic characteristics in this brain malignancy. In the current study, we investigated whether EphB2 knockdown in combination with radiation can alter invasiveness and decrease medulloblastoma tumor growth or viability in vitro.

**Methods:**

The expression of EphB2 receptor was analyzed by immunohistochemistry and Western blotting. Microarray analysis and mRNA analysis was performed on medulloblastoma patient datasets and compared to the normal cerebellum. The radiosensitization effect following EphB2 knockdown was determined by clonogenic assay in human medulloblastoma cells. Effects of EphB2-siRNA in absence or presence of radiation on cell cycle distribution, cell viability, and invasion were analyzed by flow cytometry, MTT assay, trypan blue exclusion assay, xcelligence system, and Western blotting.

**Results:**

We observed that EphB2 is expressed in both medulloblastoma cell lines and patient samples and its downregulation sensitized these cells to radiation as evident by decreased clonogenic survival fractions. EphB2 expression was also high across different medulloblastoma subgroups compared to normal cerebellum. The radiosensitization effect observed following EphB2 knockdown was in part mediated by enhanced G2/M cell cycle arrest. We also found that the combined approach of EphB2 knockdown and radiation exposure significantly reduced overall cell viability in medulloblastoma cells compared to control groups. Similar results were obtained in the xcelligence-based invasion assay. Western blot analysis also demonstrated changes in the protein expression of cell proliferation, cell survival, and invasion molecules in the combination group versus others.

**Conclusions:**

Overall, our findings indicate that specific targeting of EphB2 receptor in combination with radiation may serve as an effective therapeutic strategy in medulloblastoma. Future studies are warranted to test the efficacy of this approach in in vivo preclinical models.

**Electronic supplementary material:**

The online version of this article (doi:10.1186/s12935-017-0409-7) contains supplementary material, which is available to authorized users.

## Background

Medulloblastoma constitutes one of the most aggressive and commonly diagnosed brain malignancies in children [1]. It arises from granule neuronal precursor cells in the cerebellum or from neural stem cells of the rhombic lip [1]. The standard of care treatment for medulloblastoma is combination of surgical resection, chemotherapy, and radiation therapy [2] but tumor recurrence remains a major problem.

One of the hallmarks of medulloblastoma is rapid and extensive central nervous system (CNS) dissemination along leptomeningeal surfaces because of the presence of migratory and invasive cell populations [3]. To date, concerted research efforts have been directed in targeting these invasive and recurrent subpopulations in a tumor cell mass. However, the outcomes remain dismal. This can be attributed to the dysregulated function of some of the tyrosine kinase receptors that impart radioresistant properties and invasive potential to these tumors. For example, Morrison et al. [4], reported that certain Eph receptor tyrosine kinases and ligands were aberrantly regulated in the migrating medulloblastoma cells versus core tumor cells. These migrating cells were characterized by upregulation of EphB2 as shown by qPCR analysis in DAOY medulloblastoma cells [4]. EphB2 has also been shown to play a critical role in medulloblastoma, ependymoma, and glioblastoma (GBM) by other research groups [5–7]. For example, Sikemma et al. [5], demonstrated increased invasion in EphB2-expressing medulloblastoma cell lines following ligand stimulation. This effect was completely blocked upon shRNA-mediated knockdown of EphB2 [5]. Another group showed that EphB2 overexpression enhanced neurosphere cell migration and invasion and its targeted inhibition resulted in decreased invasion/migration in the GBM model [6].

Tumor cell migration is known to be facilitated by components such as fibronectin and collagen predominantly present in leptomeningeal extracellular matrix [8]. In addition to intrinsic promoters of cell migration such as fibronectin and collagen [8], treatment-related factors have been reported to increase tumor cell motility. Photon irradiation (IR), a standard of care in medulloblastoma treatment, has been suggested to enhance medulloblastoma cell invasion, and preclinical studies have demonstrated increased tumor cell invasiveness through upregulation of urokinase plasminogen activator receptor (uPAR) signaling [9]. Conversely, other groups have shown a decrease in migration and invasion following various dosing of photons and carbon ion irradiation [10], suggesting that additional studies are needed.

Therefore, in this study, we sought to address whether the combination of EphB2 knockdown with radiation would negate any pro-invasive effects radiation may have on medulloblastoma cells. In contrast to some observations in medulloblastoma cells [9, 11] and in non-medulloblastoma tumor cells [12], our data show that ionizing radiation did not promote tumor cell invasion but instead significantly decreased invasion, particularly in combination with EphB2 knockdown, which is consistent with other reports [10]. In addition, we found that knockdown of EphB2, combined with radiation, decreased cell viability and clonogenic survival, in part through the inhibition of cell cycle progression. This is the first study to show the impact of EphB2 inhibition in combination with radiation in medulloblastoma cells and provides a rational basis for targeted inhibition of the EphB2 receptor as a potential radiosensitization strategy in medulloblastoma.

## Methods

### Medulloblastoma patient samples and cell lines

The cohort of medulloblastoma patient samples (n = 33) was obtained from Children’s Hospital Colorado and in compliance with local and federal human research protection guidelines and Institutional Review Board regulations (COM-IRB 95-500). Out of 33 patients analyzed, there were 8 females and 25 males (Additional file 1: Table S1). Additional information on the age distribution is included in Additional file 1: Table S1. Normal cerebellum samples (n = 5) were obtained from non-malignant brain autopsies at the Children’s Hospital Colorado following Institutional Review Board guidelines. The human medulloblastoma cell lines DAOY and UW228 were obtained from the American Type Culture Collection (ATCC, Rockville, MD, USA). These cell lines were maintained in Dulbecco’s Modified Eagle’s Medium (DMEM), supplemented with 10% fetal bovine serum, and primocin (Invivogen, San Diego, CA, USA) at 37 °C and 5% CO_2_.

### RNA extraction and microarray data analysis

Total RNA was extracted from all surgical samples, amplified, labeled, and hybridized to Affymetrix plus 2 microarray chips (Affymetrix, Santa Clara, CA, USA) according to manufacturer’s instructions. Data analysis was performed in the R programming language using open-source available through Bioconductor (http://www.bioconductor.org) as described earlier [13].

### Human EphB2 mRNA expression analysis

EPHB2 expression was analyzed by using the R2 platform (http://r2.amc.nl). Specifically, the following datasets were utilized for analysis: Medulloblastoma Kool (GSE10327) [14] and Medulloblastoma Gilbertson (GSE37418) [15]. The subgroups in the Kool’s dataset are labeled based on the published literature [16, 17]. For normal expression, the Normal Cerebellum Roth dataset (GSE3526) was utilized. One-way analysis of variance was performed to compare EPHB2 expression across medulloblastoma subgroups with normal cerebellum expression.

### Immunohistochemistry

Normal human cerebellum and medulloblastoma patient sample was used to analyze the expression of EphB2 receptor by immunohistochemistry. Briefly, formalin-fixed and paraffin embedded tumor sections were deparaffinized and hydrated followed by antigen retrieval. Endogenous peroxidase activity was blocked by incubating the samples in 3% H_2_O_2_ in PBS for 30 min at room temperature. Sections were blocked in 2% milk followed by overnight incubation with anti-EphB2 antibody (5 μg/mL) at 4 °C. Anti-EphB2 antibody [18] was a kind gift by Dr. Elena B. Pasquale (Sanford Burnham Prebys Medical Discovery Institute, San Diego, CA, USA). Following washing in TBS, samples were incubated with biotinylated anti-rabbit secondary antibody for 1 h at room temperature, followed by staining with DAB reagent. Sections were counterstained with hematoxylin, and images were captured on a 20× objective using Nikon microscope.

### siRNA transfection

For transfection experiments, two different EphB2 targeting siRNAs were used. DAOY and UW228 cells were transfected in serum-free DMEM using TransIT-TKO Transfection Reagent (Mirus, Madison, WI, USA), according to the manufacturer’s instructions. Short interfering RNAs (siRNA) specific for human *EphB2* and the non-specific control siRNA (NS-siRNA) were obtained from Invitrogen (Carlsbad, CA, USA). For the functional and mechanistic experiments reported in this study, cells were transfected using 10 μL TransIT-TKO for a final working concentration of 25 nM siRNA. The transfection complex was added to the cells and 20 h post-transfection, the medium was replaced with fresh serum-containing and antibiotic-containing growth medium. Cells were analyzed at optimal time-points by different assays.

### Irradiation

Cells were irradiated with indicated radiation doses using a RS-2000 (Rad Source Technologies, Inc) X-ray irradiator, a 160 KVp source, at 25 mAmp, and at a dose rate of 1.24 Gy/min.

### Whole cell lysate preparation and immunoblotting

Medulloblastoma cells transfected with EphB2-siRNA or control NS-siRNA in the absence or the presence of radiation were harvested at different time-points. Cells were homogenized in RIPA lysis buffer (Millipore, Billerica, MA, USA), containing protease inhibitor cocktail (Thermo Fisher Scientific Inc., IL, USA) and phosphatase inhibitor (Sigma, MO, USA) on ice for 30 min. The homogenate was centrifuged at 4 °C at 13,000 rpm for 20 min, and lysates were collected. Protein concentration was determined using the BCA Protein Assay kit (Thermo Fisher Scientific Inc., IL, USA). Lysates (20–30 μg) were loaded onto 10–12% SDS-PAGE gels. Electrophoresis, blocking, probing, and detection of proteins were conducted as described earlier [19]. Membranes were probed overnight at 4 °C with respective antibodies. All primary antibodies (anti-PCNA, anti-Bcl-X_L/S_, anti-vimentin, anti-cyclinB1, and anti-β-actin) were obtained from Cell Signaling Technology (Danvers, MA, USA). Horseradish peroxidase (HRP)–conjugated secondary antibodies were obtained from Sigma (St. Louis, MO, USA).

### Clonogenic survival assay

Clonogenic survival fractions were determined following increasing doses of X-ray ionizing radiation. Cells in culture were exposed to ionizing radiation in 25 cm^3^ flasks. Clonogenic cell survival was analyzed as described [19]. Colonies comprising of at least 50 cells were counted 9–14 days post radiation treatment. After counting colonies, plating efficiency (PE) and survival fraction (SF) were determined using the formulas below:

$$ PE = Number \, of \, colonies \, formed/Number \, of \, cells \, seeded $$

$$ SF = Number \, of \, colonies \, formed \, after \, ionizing \, radiation/Number \, of \, cells \, seeded \times PE $$

Survival fraction following ionizing radiation in NS-siRNA or EphB2-siRNA transfected cells was normalized taking into consideration plating efficiency in that particular group at 0 Gy. Each experiment was replicated at least three times.

### Cell cycle analysis

DAOY cells were seeded at a density of 75,000 cells per well in six-well plates in DMEM medium containing 10% FBS and primocin. Following overnight incubation, cells were transfected using 25 nM EphB2-siRNA or control NS-siRNA in serum-free, antibiotic-free growth medium. At 24 h after transfection, the medium was exchanged with growth medium containing primocin and cells were irradiated using X-ray irradiator. At 72 h post-radiation, cells were harvested, washed in ice-cold PBS, fixed overnight in 70% ethanol, followed by staining with propidium iodide. Cell cycle distribution was analyzed at the indicated time-point by flow cytometer.

### MTT assay

DAOY and UW228 cells were seeded at a density of 150,000 cells per well in six-well plates and maintained in DMEM supplemented with 10% FBS, primocin for 24 h prior to transfection with siRNA. Cells were transfected using 25 nM EphB2-siRNA or control NS-siRNA in serum-free, antibiotic-free DMEM. At 24 h after transfection, medium was exchanged for 10% DMEM containing primocin and cells were replated at a density of 3000 cells/well in 96-well plates. After overnight incubation, cells were exposed to 8 Gy dose of radiation and analyzed 144 h later following addition of the MTT [3-(4,5-dimethylthiazol-2-yl)-2,5-diphenyltetrazolium bromide] reagent (Sigma, St. Louis, MO, USA). Optical density was measured on a microplate reader at a wavelength of 590 nm.

### Trypan blue dye exclusion

DAOY and UW228 cells were seeded at a density of 150,000 cells per well in six-well plates and maintained in DMEM containing 10% FBS and primocin. Cells were transfected as indicated above and replated at a density of 30,000 cells/well in a six-well plate. Approximately 24 h post-plating, cells were exposed to 8 Gy dose of radiation. After 120–144 h post-radiation, cells were collected by trypsinization. To each 10 μL aliquot of cells, 10 μL of trypan blue dye was added. Cell counting was performed using the T-20 automated cell counter (Bio-Rad, Hercules, CA, USA).

### Invasion assay

The xCELLigence RTCA DP Analyzer (Roche, CA) was used to determine the effect of EphB2 knockdown in absence and presence of radiation on invasion in a CIM-16 plate (ACEA Biosciences, San Diego, CA, USA). The CIM-16 plate is a modified Boyden chamber in which the porous membrane separating the double-chambered well is coated with gold microelectrodes. In this system, cells invading through the upper chamber into the lower chamber adhere to these microelectrodes, increasing the electrical impedance across this membrane. Electrical impedance is measured as a “cell index,” which directly correlates to the number of cells that have invaded into the lower chamber and adhered to the underside of the membrane. Background measurement was taken by adding cell-free, serum-free DMEM to the upper and lower chambers of each well. The plate was incubated at 37 °C for 1 h prior to measuring background electrical impedance for each well. At 20 h post-radiation, cells were starved for 4 h in serum-free, and antibiotic-free DMEM, prior to collection by trypsinization. Cells were resuspended in serum-free DMEM with primocin and seeded in the upper chamber (coated with 1:40 dilution of matrigel) at a density of 50,000 cells per well. Invasion was monitored for 35 h. Delta cell index values were calculated by subtracting cell index values in the absence of a serum gradient from cell index values in the presence of a serum gradient. Cell index values between different treatment groups were determined at 3 h-intervals after the start of the experiment.

### Statistical analysis

All the experiments were performed in duplicate or triplicate and repeated two to three times. Quantitative analyses were performed using Student’s t test or one way ANOVA by GraphPad Prism software. A p value of <0.05 was considered significant.

## Results

### Medulloblastoma patient samples express high levels of EphB2 receptor

**Fig. 1:**
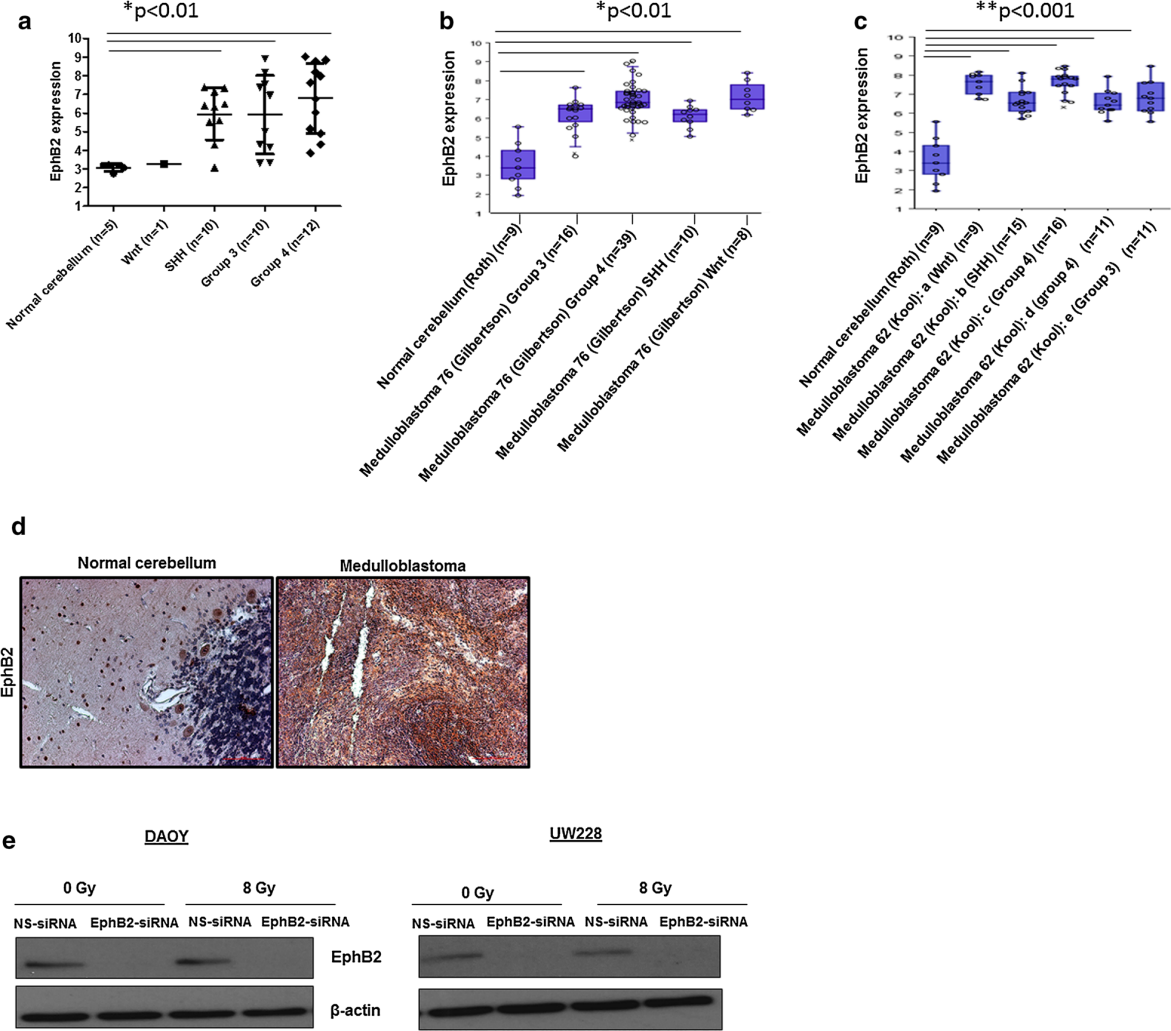
EphB2 is expressed in both medulloblastoma patient samples from different medulloblastoma subtypes and cell lines. **a** Transcriptomic data analysis show that EphB2 receptor is overexpressed in four distinct medulloblastoma subtypes compared to normal counterparts. Similar trend was evident with the mRNA analysis performed on EPHB2 expression by using the R2 platform (http://r2.amc.nl). The Medulloblastoma Gilbertson (GSE37418) dataset (**b**) and Kool (GSE10327) dataset (**c**) were used for the analysis [15]. For normal expression the Normal Cerebellum Roth dataset (GSE3526) was utilized. The subgroups in the Kool’s dataset are labeled based on the published literature [16, 17]. One-way analysis of variance was performed to compare EPHB2 expression across medulloblastoma subgroups with normal cerebellum expression. **d** IHC analysis show that EphB2 is expressed at high levels in medulloblastoma patient samples compared to the normal cerebellum. **e** EphB2 expression is decreased following transfection with EphB2-specific siRNA alone and in the presence of 8 Gy dose of radiation compared to control non-specific siRNA (NS-siRNA) at 72 h post-XRT

Morrison et al. [4], reported significant upregulation in transcript levels of the EphB2 receptor in migrating medulloblastoma cells. Based on recent studies, medulloblastoma can be categorized into four distinct molecular subtypes: Wnt, Sonic Hedgehog (SHH), Group 3, and Group 4 [16, 17]. We analyzed transcriptomic data performed on a cohort of medulloblastoma patients obtained from Children’s Hospital Colorado and found that EphB2 is significantly overexpressed in these samples compared to the non-malignant brain samples (Fig. 1a). We also performed mRNA analysis on the two different datasets: Medulloblastoma Kool (GSE10327) [14] and Medulloblastoma Gilbertson (GSE37418) [15]. Although variability can be seen across the different datasets, similar trends were observed with all the four medulloblastoma subtypes showing elevated expression of EphB2 compared to the normal cerebellum (Fig. 1b, c). We next validated these data by analyzing the expression of EphB2 receptor in medulloblastoma patient samples by immunohistochemistry. Normal cerebellum was used as a negative control. As shown in Fig. 1d, higher EphB2 levels are present in medulloblastoma patient samples as compared to the normal cerebellum.

### EphB2 receptor is efficiently knocked down using targeted siRNA approach in medulloblastoma cells

We validated the expression of EphB2 in two different human medulloblastoma cell lines DAOY and UW228. The DAOY cell line is derived from a 4-year old male patient and the UW228 cell line is derived from a 9-year old female patient [20]. The EphB2 receptor is expressed in both DAOY and UW228 cells (Fig. 1e). To investigate the functional role of EphB2 in our in vitro model system, we transiently transfected medulloblastoma cells with either an EphB2-siRNA or a control NS-siRNA and analyzed the knockdown efficiency at protein level. Our Western blot analyses show a reduction in EphB2 levels in the cells transfected with EphB2-specific siRNA compared to control NS-siRNA at 72 h post-transfection (Fig. 1e).

### EphB2 receptor knockdown sensitizes medulloblastoma cells to ionizing radiation

**Fig. 2:**
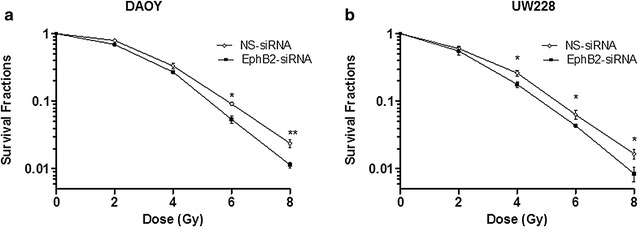
EphB2 knockdown radiosensitizes medulloblastoma cells to ionizing radiation. Clonogenic assay show reduction in survival fractions in DAOY cells (**a**) and UW228 cells (**b**) following transfection with EphB2-targeting siRNA (25 nM) versus control NS-siRNA (25 nM) at increasing doses of radiation. Each clonogenic assay was repeated at least three times. Representative survival plots are shown for each cell line. Data shown represent mean ± standard deviation. Student’s t test was used to compare point-by-point differences between NS-siRNA and EphB2-siRNA transfected group.*p < 0.05, **p < 0.005

Clonogenic survival assays were performed to determine whether knockdown of EphB2 receptor can enhance radiosensitization in medulloblastoma cells. EphB2-siRNA or NS-siRNA transfected cells were subjected to increasing doses (0, 2, 4, 6, and 8 Gy) of ionizing radiation. After 9–14 days of radiation treatment, we analyzed the clonogenic survival fractions in both experimental and control groups. Our data demonstrate that following EphB2 knockdown, both DAOY and UW228 cells became more sensitive to ionizing radiation (Fig. 2a, b). Analysis of the survival fractions in DAOY cells show that SF6 (survival fraction at 6 Gy dose of ionizing radiation) values were decreased from 0.09 in the NS-siRNA control group to 0.05 in the EphB2 knockdown group (Fig. 2a). In UW228 cells, the SF6 values were reduced from 0.06 in NS-siRNA transfected group to 0.04 in the EphB2 siRNA transfected group (Fig. 2b).

### EphB2 receptor knockdown followed by radiation enhances cell cycle distribution in G2/M phase in medulloblastoma cells

**Fig. 3:**
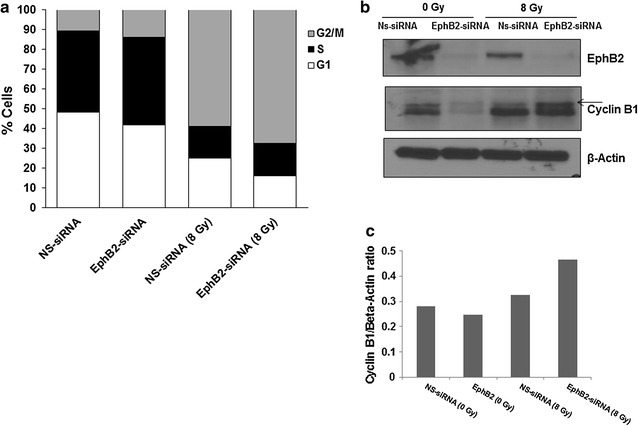
EphB2 knockdown combined with ionizing radiation results in G2/M arrest in medulloblastoma cells. **a** Increased accumulation of DAOY cells in G2/M phase of the cell cycle is evident in flow cytometry analyses following combined EphB2 knockdown and ionizing radiation. The cells were analyzed at 72 h post-radiation. The experiment was repeated at least two to three times. Representative plot is shown for DAOY cell line. **b** An increase in the expression of CyclinB1 is observed in DAOY cells transfected with EphB2-specific siRNA and exposed to 8 Gy dose of radiation. **c** Densitometric analysis was performed to quantify Cyclin B1 expression across different treatment and control groups

After observing the EphB2-knockdown mediated radiosensitization effects in vitro, we investigated the underlying mechanism by which knockdown of EphB2 enhanced radiosensitivity of medulloblastoma cells. Cell cycle analyses were performed by flow cytometry to determine the combined effects of EphB2 knockdown and radiation on the cell cycle distribution. The DAOY cells transfected with EphB2-siRNA and exposed to radiation were predominantly arrested in the G2/M phase of cell cycle as depicted by an increase in percentage of cells (approx. 67%) in this phase compared to other treatment groups (Fig. 3a). Further, Western blot analysis confirmed an increase in CyclinB1 protein levels in the EphB2-siRNA transfected cells followed by radiation as compared to either NS-siRNA or EphB2-siRNA transfected groups (Fig. 3b), indicative of arrest in the late G2 or the M phase of the cell cycle. Densitometric analysis of CyclinB1 expression is shown in Fig. 3c.

### Combined EphB2 receptor knockdown with radiation decreases medulloblastoma cell growth and viability

**Fig. 4:**
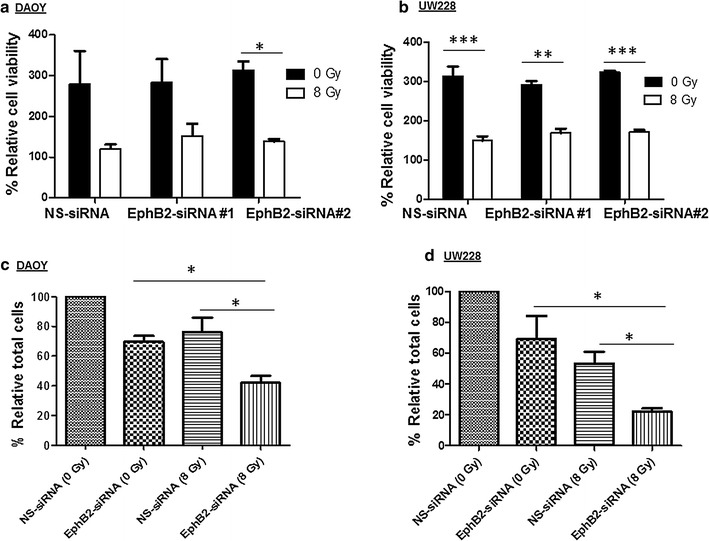
EphB2 knockdown when combined with radiation treatment results in decreased cell viability in medulloblastoma cells. MTT analysis (**a**, **b**) and Trypan blue analysis (**c**, **d**) show significant decrease in cell viability and decrease in relative percent of total number of cells in both DAOY and UW228 cells when knocked down for EphB2 receptor and combined with 8 Gy dose of radiation compared to other control and experimental groups at 120–144 h post-XRT. Each experiment was replicated two to three times. Data represent mean ± standard error. Student’s t test was used to make comparisons between different groups. *p < 0.05, **p < 0.005, ***p < 0.0005

We determined the effect of EphB2 knockdown on cell viability by performing both MTT and trypan blue exclusion assays. DAOY and UW228 cells that were knocked down for EphB2 receptor did not show any significant change compared to control siRNA transfected group (Fig. 4a, b). However, when the EphB2-siRNA transfected cells were combined with an optimal dose of radiation, a significant decrease in cell viability was observed in both the medulloblastoma cell lines (Fig. 4a, b). Similar to MTT results, trypan blue data showed significant decrease in total number of cells in the EphB2 knockdown group followed by radiation exposure compared to EphB2 knockdown alone or control NS-siRNA group in both DAOY and UW228 cells (Fig. 4c, d). In DAOY cells, the relative percent of total number of cells decreased to ~40% in the combination group compared to ~70–75% in the single agent treatments (Fig. 4c). In UW228 cells, the relative percent of total number of cells declined to ~20% in the combination group compared to ~60–70% in the single agent treatments (Fig. 4d).

### EphB2 knockdown combined with radiation decreases invasion in medulloblastoma cells

**Fig. 5:**
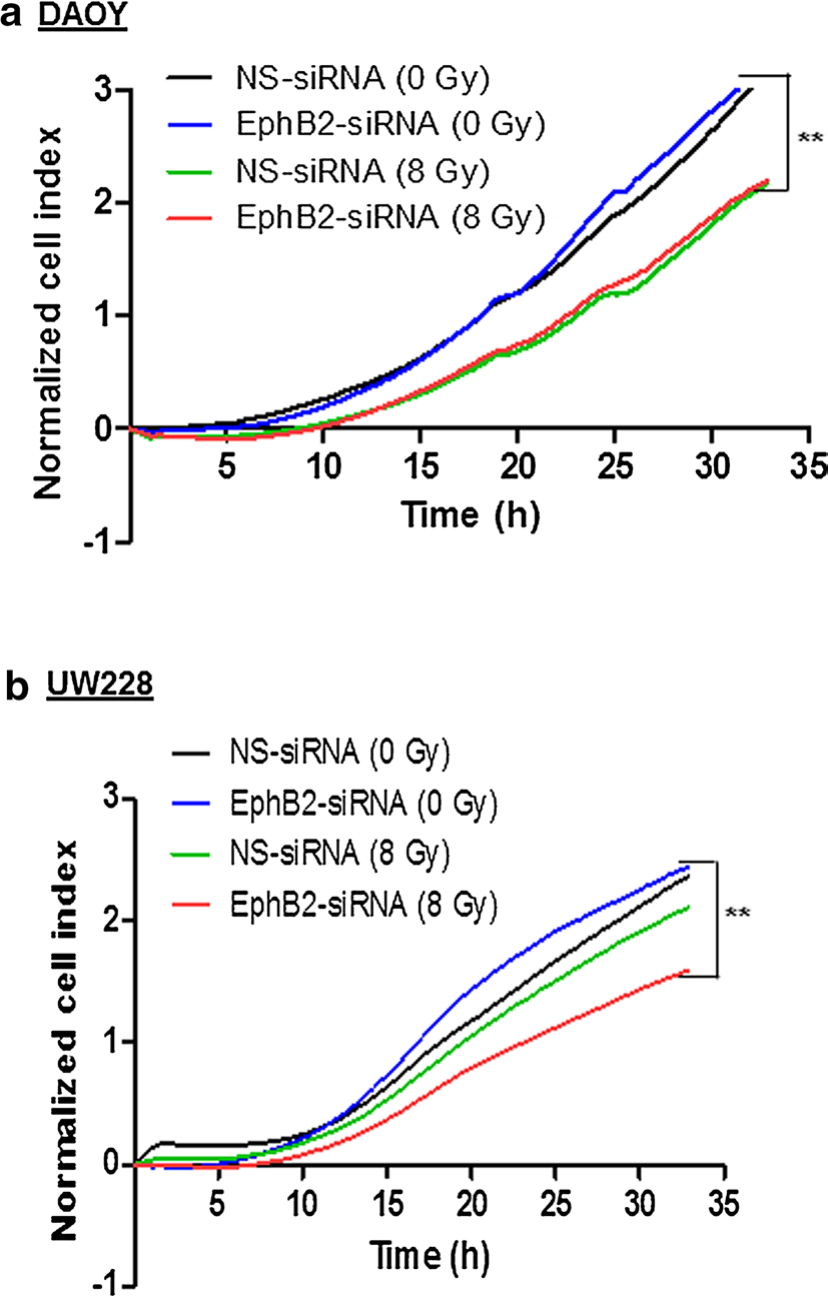
EphB2 knockdown in combination with ionizing radiation results in decreased cell invasion in medulloblastoma cells. EphB2 knockdown when combined with radiation significantly decreases cell invasion compared to non-irradiated groups in DAOY (**a**) and UW228 (**b**) cells. Cell invasion was measured in a transwell chamber using electrical impedance method. Cell index values represent changes in electrical impedance across the membrane separating the upper and lower chambers, and show a direct correlation with the number of cells that have invaded into the lower chamber. Background invasion was subtracted to obtain normalized cell index values. Representative plot is shown in this figure for each individual cell line. Each experiment was repeated at least two times. **p < 0.005

We next investigated the effect of EphB2 knockdown followed by radiation on medulloblastoma cell invasion in an electrical impedance-based assay. In DAOY cells, although we did not notice any significant change in invasion in the EphB2-siRNA transfected cells compared to the NS-siRNA group, combined EphB2 knockdown and radiation resulted in a significant reduction in invasion compared to non-irradiated groups (Fig. 5a). We observed a similar trend in UW228 cells. When EphB2-siRNA transfected cells were exposed to radiation, a significant decrease in invasive ability of UW228 cells was observed as evident by the decrease in cell index values compared to either NS-siRNA or EphB2-siRNA alone (Fig. 5b).

### EphB2 knockdown with radiation modulates the expression of cell proliferation, invasion, and survival proteins

**Fig. 6:**
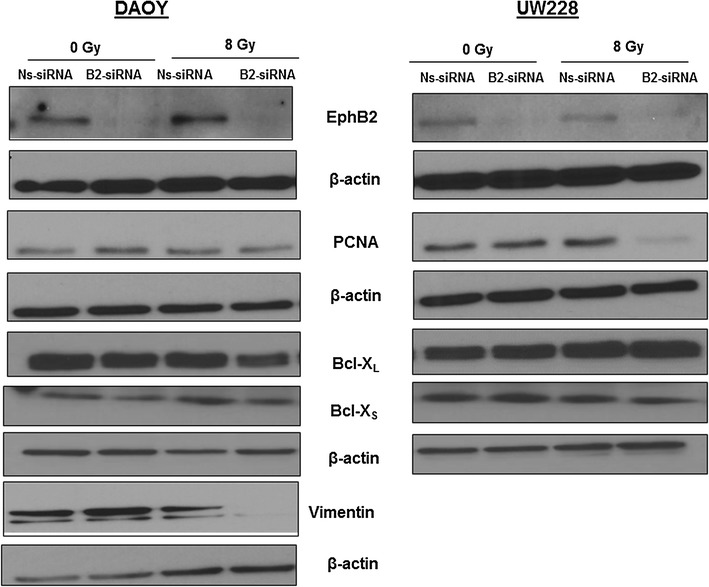
EphB2 knockdown with ionizing radiation results in altered expression of cell survival, proliferation, and invasion related proteins in medulloblastoma cells. UW228 cells show decreased expression of PCNA, a proliferation marker, in the combined treatment group compared to other groups but is reversed to normal baseline levels in the DAOY cell line. Bcl-X_L_, which is a pro-survival marker, also shows marked reduction in the EphB2-siRNA transfected and irradiated group in DAOY cells. Bcl-X_S_, is slightly reduced in the combination group in UW228 cells. Vimentin, a marker that plays a key role in invasion, similarly shows marked reduction when DAOY cells knocked down for the EphB2 receptor were exposed to 8 Gy dose of radiation. The analysis was performed 24 h post-XRT for all the markers except for Bcl-X_L_ and Bcl-X_S_ where analysis was performed at 72 h post-XRT

To identify the proteins associated with the effects observed following EphB2 knockdown in the presence of radiation, Western blot analyses were performed. Our data demonstrate a decrease in the levels of PCNA, a surrogate marker for proliferation, in both DAOY and UW228 cells following combined treatment with EphB2 knockdown and radiation compared to other groups (Fig. 6). The decrease was more prominent in UW228 cells. In addition, the levels of Bcl-X_L_, a pro-survival marker, were also altered in the combination group in DAOY cells (Fig. 6). In UW228 cells, Bcl-X_S_ show a slight reduction when the EphB2 knockdown cells were exposed to 8 Gy dose of radiation compared to other groups (Fig. 6). We also observed changes in the levels of proteins such as vimentin that impart invasive characteristics to the cancer cells. The level of vimentin was decreased in DAOY cells following EphB2 knockdown and radiation compared to other experimental or control groups (Fig. 6).

## Discussion

Medulloblastoma comprises of one of the most frequently occurring solid malignancies and leading cause of cancer related deaths in children. Based on the molecular profiling, medulloblastomas are categorized into four principal subgroups: Wnt, Sonic hedgehog (SHH), Group 3, and Group 4 [16, 17, 21]. The classification of these subgroups is based on the specific signaling pathways thought to be dysregulated in their pathogenesis and by clinical, histological, and genetic features [17]. Currently, treatment strategies for medulloblastoma patients are largely influenced by factors such as risk stratification and age of the patient. Recent subclassification and exploration into the particular pathways and targets predominantly affected in each of these subgroups may allow personalized management of these tumors. In the analysis performed on medulloblastoma patients, we observed a variable but significant increase in the expression of EphB2 across the different subgroups compared to the normal cerebellum. Growing evidence over the past few years have reported aberrant regulation of the Eph family of receptors and their cognate ligands in a number of human malignancies, including medulloblastoma [4–6, 19, 22–24]. In the present study, we investigated, for the first time, the effects of combined EphB2 knockdown and radiation on cell growth, viability, and invasiveness of medulloblastoma cells. Our data show that the combined downregulation of EphB2 and radiation exposure decreased clonogenic survival fractions, induced G2/M cell cycle arrest, inhibited cell growth and viability, and reduced cell invasion. These effects were mainly studied in two medulloblastoma cell lines DAOY and UW228, that are reportedly associated with the SHH subgroup [25, 26].

We documented, for the first time, that EphB2 receptor knockdown sensitizes medulloblastoma cells to ionizing radiation. It is consistent with previous reports showing that in addition to stimulating tumor growth, development, and metastasis, Eph/ephrin family members also confer radioresistant characteristics to cancer cells [19, 27]. Our cell cycle data show an increase in the accumulation of cells in G2/M phase when EphB2-siRNA transfected cells were exposed to an optimal dose of radiation. Considering that G2/M phase is one of the most radiosensitive phase of the cell cycle, our data is consistent with the published literature [28].

Following cell cycle distribution analysis, we also performed cell viability assays demonstrating growth inhibitory effects of EphB2 knockdown when combined with radiation. EphB2 knockdown alone did not produce any significant changes in cell viability in both DAOY and UW228 cells. Our data are consistent with a study published by Sikemma and colleagues where they did not observe any direct effects on cell proliferation in medulloblastoma cell lines following silencing of EphB2 receptor [5]. However, EphB2 knockdown approach in combination with radiation, resulted in a significant reduction in viability as observed in both the medulloblastoma cell lines. This synergy is further validated in clonogenic assay where EphB2 knockdown enhances the radiosensitivity of these cells that appears to be partially mediated via cell cycle arrest. In line with our functional assays, we observed changes in the expression of proteins that play a critical role in tumor cell growth, proliferation, invasion, and cell survival pathways in combined EphB2 downregulated and irradiated group versus other groups.

Medulloblastoma tumors are characterized by the presence of tumor cell populations with high invasive potential, facilitating migration of these cells along leptomeningeal surfaces [3]. It has been reported that medulloblastoma cells with high migratory phenotype exhibit elevated levels of EphB2 receptor [4] that could be responsible for increased migration/invasion. Sikemma et al. [5], also demonstrated enhanced effects of ligand-mediated stimulation of EphB2 receptor on medulloblastoma cell invasion, which was further abrogated following shRNA mediated silencing of EphB2 receptor. Similar results were observed by another group in a GBM model, where EphB2 gain-of-function experiments increased neurosphere cell migration and invasion [6]. In addition to EphB2, other studies in the literature have shown that medulloblastoma cells when exposed to radiation exhibit enhanced cell invasion [9, 11]. Therefore, in our study, we addressed the question whether knockdown of EphB2 receptor in combination with radiation would decrease the pro-invasive characteristics of medulloblastoma cells. Our findings show that when DAOY and UW228 cells were exposed to radiation, it resulted in decreased cell invasion. This is consistent with the findings published by Rieken et al. [10]. The key observation in our experiments is the significant reduction in invasive potential of medulloblastoma cells following combined inhibition of EphB2 knockdown with radiation compared to other groups. This suggests that while EphB2 knockdown by itself may not be sufficient to produce desirable result and that inhibiting the expression or function of EphB2 could be beneficial when used as part of a radiosensitization strategy to reverse the malignant and invasive phenotype.

## Conclusions

In conclusion, our data shed light on the efficacy of strategy involving combined EphB2-knockdown with radiation in medulloblastoma. This is evident by alterations in clonogenic survival, cell cycle distribution, medulloblastoma cell viability, and invasive potential in vitro. Future studies are warranted to test the efficacy of combined modality described in this study in other pre-clinical models. Regardless, our results emphasize the importance of combined EphB2 downregulation with radiation as a potential therapeutic approach to achieve anti-tumorigenic effects in pediatric medulloblastoma.

## Additional file

**Additional file 1.** Patient information on age and sex distribution.
