# Supplementary figures and images for: Combined EphB2 receptor knockdown with radiation decreases cell viability and invasion in medulloblastoma
Source: Cancer Cell Int. 2017 Mar 29;17:41. doi: 10.1186/s12935-017-0409-7 (PMC5371267; doi:10.1186/s12935-017-0409-7)

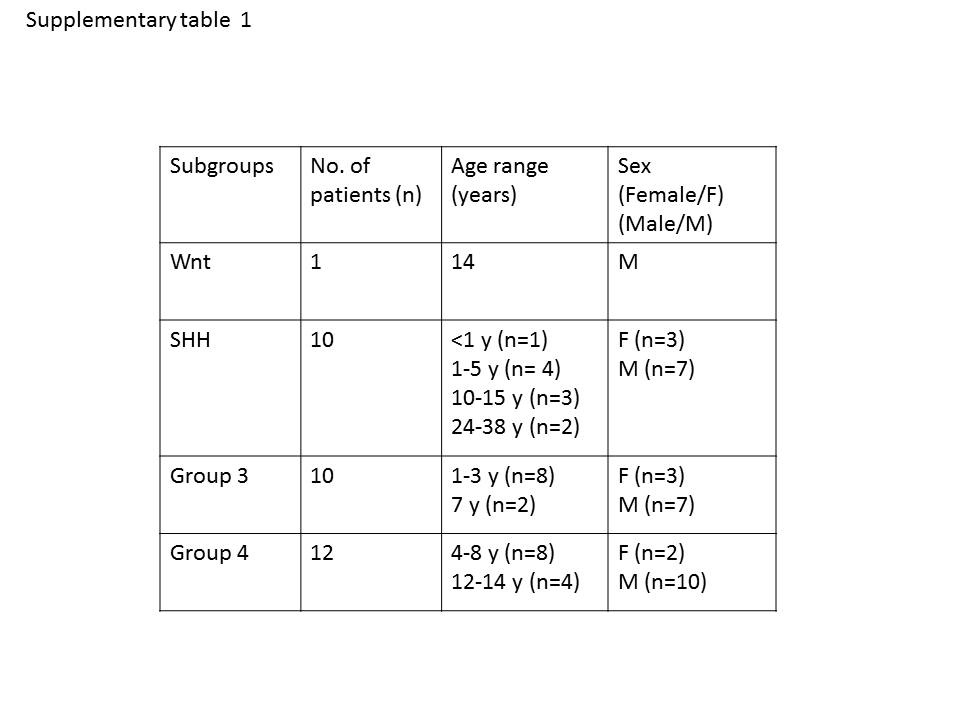

Supplement: Supplementary file 1 — Additional file 1. Patient information on age and sex distribution. [file 12935_2017_409_MOESM1_ESM.tif]
